# Voluntary activation failure is detectable in some myositis patients with persisting quadriceps femoris weakness: an observational study

**DOI:** 10.1186/ar1935

**Published:** 2006-04-10

**Authors:** Catherine B Molloy, Ahmed O Al-Omar, Kathryn T Edge, Robert G Cooper

**Affiliations:** 1 University of Manchester Rheumatic Disease Centre, Hope Hospital, Eccles Old Road, Salford M6 8HD, UK

## Abstract

This cross-sectional, observational study was undertaken to examine whether voluntary activation failure could contribute to the persisting weakness observed in some patients with treated idiopathic inflammatory myositis. In 20 patients with myositis of more than six months' duration (5 males, 15 females; mean [± 1 SD] age 53 [11] years) and 102 normal subjects (44 males, 58 females; mean age 32 [8] years), isometric maximum voluntary contractions (MVCs) of the dominant quadriceps femoris (QF) were quantified. Absolute MVC results of normal subjects and patients were then normalised with respect to lean body mass (force per units of lean body mass), giving a result in Newtons per kilogram. Based on mass-normalised force data of normal subjects, patients were arbitrarily stratified into "weak" and "not weak" subgroups. During further MVC attempts, the "twitch interpolation" technique was used to assess whether the QF voluntary activation of patients was complete. This technique relies on the fact that, because muscle activation is incomplete during submaximal voluntary contractions, electrical stimulation of the muscle can induce force increments superimposed on the submaximal voluntary force being generated. No between-gender differences were seen in the mass-normalised MVC results of healthy subjects, so the gender-combined results of 6.6 (1.5) N/kg were used for patient stratification. No between-gender difference was found for mass-normalised MVCs in patients: males 5.4 (3.2) and females 3.0 (1.7) N/kg (*p *> 0.05). Mass-normalised MVCs of male patients were as great as those of normal subjects (*p *> 0.05), but mass-normalised MVCs of female patients were significantly smaller than those of the normal subjects (*p *< 0.001). Only one of the six "not weak" patients exhibited interpolated twitches during electrical stimulation, but six of the 14 "weak" patients did, the biggest twitches being seen in the weakest patient. That interpolated twitches can be induced in some myositis patients with ongoing QF weakness during supposed MVCs clearly suggests that voluntary activation failure does contribute to QF weakness in those patients.

## Introduction

Polymyositis (PM) and dermatomyositis (DM) are the idiopathic inflammatory myositis subtypes most often treated by rheumatologists [1,2]. Corticosteroids and immunosuppressive drugs remain the mainstay of treatment [2], but the response to these agents is often disappointing, so chronic weakness and disability may persist despite treatment [3,4]. In chronic, end-stage myositis, in which muscle wasting may be radiologically and even clinically obvious, weakness may be explained by loss of muscle mass which, once established, often appears irreversible. In the early acute phase of myositis, when muscle histology might demonstrate the characteristic infiltration by T cells and macrophages, with secondary muscle fibre damage and myonecrosis [5], weakness is often at its most severe. Because early weakness usually improves with treatment, albeit to a variable degree, it has traditionally been assumed that muscle weakness prior to treatment results from inflammatory processes, although the actual mechanisms responsible for inflammatory weakness induction remain unelucidated. In treated myositis, recovery of strength is often incomplete, even though radiological and histological evidence suggests that inflammation has been suppressed. Here, especially in the absence of obvious wasting, persisting weakness is harder to explain. Thus, it is also increasingly recognised in myositis that any correlation between the observed weakness of a muscle and the degree of its inflammatory cell infiltration at biopsy may be very poor [6,7]. These discussions clearly suggest that mechanisms other than those related to inflammation are implicated in weakness induction in myositis [8]. Indeed, many recent studies present compelling evidence that abnormalities of energy metabolism [9,10], possibly due to disruption of local microcirculation [11], as well as cytokine dysfunction [12-14], are likely involved in weakness induction. More recently still, in murine and human myositis, activation of the endoplasmic reticulum stress response has been demonstrated as another, self sustaining, nonimmune mechanism capable of inducing skeletal muscle cell dysfunction and loss in myositis [15]. These nonimmune myositis-induced abnormalities would all likely cause muscle weakness by disrupting contractile function.

Although the importance of these recent findings in terms of understanding nonimmune-mediated weakness induction in myositis is obvious, there are other mechanisms that may be important and that have not been investigated to date. Skeletal muscle weakness can result from a defect at any step in the neuromuscular command chain governing contraction [16]. In general, however, weakness is termed "central" if due to a defect prior to the neuromuscular junction or "peripheral" if due to a defect beyond the junction [16]. Applying such principles in myositis, inflammatory damage to the muscle membrane and contractile apparatus itself would obviously cause peripheral dysfunction. Abnormalities of the spinal cord, anterior horn cells, and peripheral nerves are not part of the usual clinical spectrum of myositis, so all appear unlikely causes of central dysfunction. However, other central factors such as insincerity of effort due to poor motivation/perceived illness, or pain inhibition due to myalgia, could theoretically contribute to weakness in myositis. In addition, reflex inhibition is a possibly relevant mechanism. It is well recognised clinically that acute knee joint pathologies cause rapid quadriceps femoris (QF) weakness and wasting. It has also been shown that acute iatrogenic knee joint effusions cause QF weakness [17,18], which can be prevented if the joint is rendered insensate by local anaesthetic co-injected with the iatrogenic effusion [19]. It was thus concluded that acute joint knee pathologies, including iatrogenic effusions, cause QF weakness by stimulating joint afferents, which reflexively inhibit anterior horn cell function and thereby reduce QF motor activation and cause "arthrogenous" QF weakness [20]. In an analogous fashion, it seems theoretically plausible in myositis that inflammatory cell infiltrates could stimulate muscle afferents and similarly inhibit anterior horn cell function. The result would be "myogenous" weakness. The possibility that central activation failure from motivational problems and/or reflex inhibition could be responsible for weakness induction in myositis has never been assessed. This study of patients with myositis was therefore undertaken to examine the completeness of central activation during maximum voluntary contractions (MVCs) of QF.

## Materials and methods

### Patients with myositis

Twenty patients (15 females and 5 males) with adult (onset at or after 18 years) myositis, defined as definite according to the Bohan and Peter diagnostic criteria [21], were recruited into this observational, cross-sectional study, which was approved by the local ethics committee. Nine patients had PM, 5 had DM, and 6 had PM as part of a connective tissue disease overlap. Their mean age (± 1 SD) was 53 (11) years, and their mean myositis disease duration was 5.6 (3.9) years (Table 1). Current disease status of patients was assessed using the standard clinical tools available in the outpatient setting (that is, the results of "extended" manual muscle testing [MMT] [22] and circulating creatinine kinase [CK] and C-reactive protein [CRP] levels). Lower limb magnetic resonance imaging (MRI), QF muscle biopsy by conchotome [23], and needle electromyography (EMG) were not specifically used in this study to assess disease activity, but if any of these procedures had been undertaken for clinical reasons in the previous six months, their results were obviously used during disease assessments. According to the results of these clinical parameters, and based on "intention to treat" principles, patients' myositis disease activity and damage status was "guestimated" (Table 2). This simple scoring system was used because, although international efforts to develop comprehensive disease activity and damage assessment tools are in an advanced state of development [24,25], work validating these tools is still ongoing [26,27], and international consensus on their final versions is awaited. Patients' clinical details and disease activity and damage guestimates at the time of their recruitment are summarised in Tables 1 and 3. Patients suffering current QF myalgia were excluded because this could have caused weakness through pain inhibition. Patients with symptoms or signs of knee joint pathologies, such as osteoarthritis, that could cause arthrogenous QF weakness were also excluded.

### Normal subjects

Forty-four normal males (32.4 [7.9] years old) and 58 normal females (28.5 [6.8] years old) were recruited from hospital medical and nonmedical staff. Considerable efforts to age-match these subjects with the myositis patients were made, but older staff proved difficult to recruit; as a result, the normal subjects were significantly younger than the patients (*p *< 0.001). However, this age difference was not considered problematic, because it was known already from MMT results that many of the patients were weak, and the rationale for testing normal subjects was not to make direct comparisons with patients regarding QF force results. Instead, the aim of using normal data was to set a mass-normalised QF MVC limit below which patients' results could arbitrarily be defined as "weak" or "not weak." As with patients, normal subjects were excluded if they had any symptoms or signs of knee joint pathologies.

### Measuring lean body mass, QF MVC, and mass-normalised MVC

In patients and normal subjects, lean body mass (LBM) was derived from skin-fold thickness measurements using well validated methods [28] before MVCs of their dominant QF muscle were measured on a standard isometric strain-gauge test-chair, based on the design of Edwards and Hyde [29,30]. Because MVCs are rarely used during normal daily activities, patients and normal subjects were first familiarised with MVC force generation. To avoid subsequent fatigue effects on the test day results, this was undertaken 1 week prior to formal MVC testing. During MVC testing, subjects sat upright on the test chair, with knees and hips set at 90 degrees. An inextensible band, velcro-fastened securely around the ankle proximal to the malleoli, connected subjects to the force transducer. The transducer output was amplified and simultaneously recorded on a chart recorder, and a custom-built monitor displayed the attained force in Newtons. A restraining belt was also velcro-secured around test subjects' waists during their efforts to generate MVCs to minimise any test-induced change in hip angle. During MVCs, subjects received vigorous verbal encouragement to perform maximally, as well as visual feedback via the monitor displaying their attained force and the chart recorder output. Contraction attempts of 3–5 seconds were made, 1 minute apart, until MVCs were within 5% to 10% of each other, which is accepted as MVC in normal subjects under these conditions [29]. With prior familiarisation and with verbal and visual feedback, all normal subjects attained their MVC within three attempts on the test day. The test technique and feedback given were identical for patients, none of whom complained of QF myalgia prior to, during, or after MVC testing.

Though a close relationship has been established between body mass (BM) and absolute QF MVC results, the expected force at any BM may range widely (for example, at 70 kg – the mean [± 1 SD] QF MVC force is 350 [70] N) [29], thus making between-subject comparisons problematic. To overcome this problem, MVC results of normal subjects and patients were mass-normalised. Thus, once each normal subject's and patient's LBM had been determined, their absolute QF MVC force was divided by their LBM, giving a mass-normalised MVC result in Newtons per kilogram.

### Twitch interpolation

**Figure 1:**
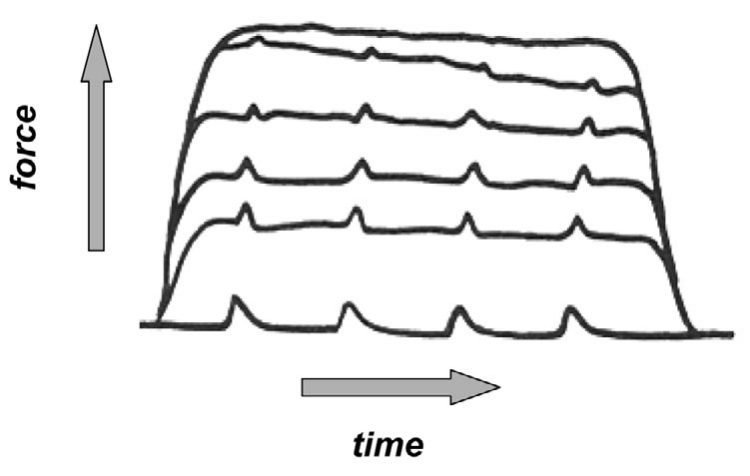
The effect of superimposed twitches on incremental voluntary quadriceps femoris (QF) contractions using the twitch interpolation technique. During a voluntary contraction, 1-Hz electrical twitches are delivered via surface electrodes applied over the motor nerve or muscle motor points. At low levels of voluntary activation (on the y-axis), a large proportion of muscle fibres remain unactivated, so that superimposed stimulation can induce large interpolated twitches. As the level of voluntary activation increases, the proportion of yet unactivated fibres decreases and so the height of the stimulation-induced interpolated twitches decreases until, near or at maximum voluntary contraction (MVC), twitches can no longer be seen (adapted from [32]).

Whether or not a muscle is being maximally activated voluntarily can be assessed using the "twitch interpolation" technique [31-34]. The basis of this is that, if a muscle is being submaximally activated voluntarily, further activation is possible by superimposing electrical stimulation via surface electrodes applied over the motor nerve or over the muscle motor points. If voluntary activation is not complete, such stimulation will produce force increments over and above the submaximal voluntary force being generated [32] (Figure 1).

If interpolated twitches are detected during "supposed" MVCs (as has been observed, for instance, in chronic fatigue syndrome [35] and chronic fibromyalgia syndrome [36] patients), this confirms that voluntary activation is not complete.

It is known from previous use of the twitch interpolation technique that normal individuals, with prior familiarisation and visual feedback, can reliably attain MVCs [32,37,38], so interpolated twitches were not deemed necessary here to prove maximal voluntary QF activation in normal subjects. For twitch interpolation in patients, two flexible 16 × 12 cm carbon-impregnated silicon equine electrodes (Henleys Medical Supplies Ltd., Welwyn Garden City, Hertfordshire, UK) were applied proximally and distally to the lateral and medial thigh, respectively (that is, over the proximal and distal QF motor points). Electrical contact was optimised with a highly conductive electromedical gel (Dracard electrode gel; Crown Graphic Ltd., Totnes, Devon, UK). The electrode placements were secured by a bandage wrapped around the thigh, immediately prior to the patients' being seated on the MVC test-chair. Electrical stimulation (Devices 3072; Digitimer Ltd, Welwyn Garden City, Hertfordshire, UK) was computer-controlled (Amstrad PC1640; Amstrad Plc, Brentwood, Essex, UK) by specifically written software (Programmable Stimulator Controller, PULG10 Rev 1.2; Computer Allied Services, Queensland, Australia) and delivered at 1 Hz. In previous interpolated twitch studies in normal subjects, supramaximal stimulation voltages were usually used [33,39,40], but many of our patients with myositis appeared intolerant of such intense stimulation, so the voltages used instead were ones that were easily tolerated by them all. After familiarisation with twitches at clearly submaximal voltages, all patients eventually tolerated stimulation voltages of 90–100 V, and we were able to increase the square-wave pulse width from 50 to 500 μs at these voltages, which were then used during interpolation testing.

Having determined each patient's supposed MVC, stimulated twitches at test voltages were then delivered to the resting QF, for further familiarisation purposes. After a 60-second rest without stimulated twitches, these were restarted, and patients were asked to perform QF contractions at approximately 50% of their previously attained MVC, aided by visual feedback from their force traces, on which superimposed twitches were then visualised. After another 60-second rest without twitches, these were again restarted at rest and patients were then asked to produce another 3- to 5-second MVC, with full verbal and visual feedback, as already described. Voluntary QF activation was deemed incomplete if, during these supposed MVCs, interpolated twitch-induced force increments could be seen on the voluntary force trace.

### Statistical methods

The results are shown as the mean (± 1 SD). Between-group comparisons were performed using χ^2 ^and Mann-Whitney *U *tests. Pearson coefficients (*r*) were derived for correlation analysis.

## Results

The patients with myositis were significantly older and heavier than the normal subjects: 53 (11) *vs. *30 (8) years and 77.3 (13.2) *vs. *70.5 (12.9) kg, respectively (*p *< 0.001 and < 0.02, respectively). As was expected from their larger masses, normal males were significantly stronger than normal females in absolute terms (that is, their MVCs were 388.5 (102.1) *vs. *304.8 (82.5) N, *p *< 0.001). However, no between-gender differences were seen for mass-normalised MVC, with MVC/LBM results of 6.4 (1.3) and 6.8 (1.6) N/kg for normal males and females, respectively (*p *> 0.05). Thus, their gender-combined results of 6.6 (1.5) N/kg were used during subsequent patient stratification (see below). With an MVC/LBM of 5.4 (3.2) N/kg, male patients were, as a group, as strong as normal males (*p *> 0.05). With an MVC/LBM result of 3.0 (1.7) N/kg, female patients were as strong as male patients (*p *> 0.05) but significantly weaker than the gender-combined normals (*p *< 0.001).

**Figure 2:**
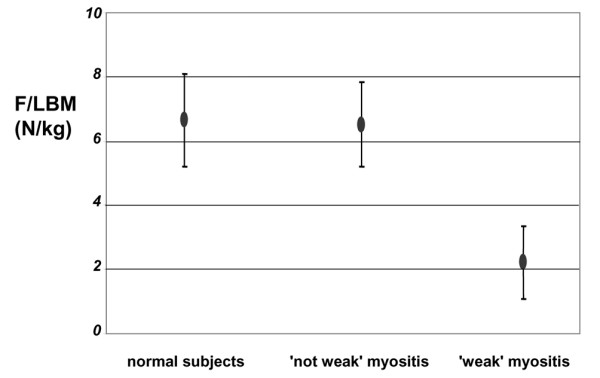
Mass-normalised force results for normal subjects and the "not weak" and "weak" patients with myositis. Males and females are combined in all three groups. The "weak" patients with myositis were obviously weak relative to the other groups, with a mass-normalised force result of 2.2 N/kg compared with 6.6 N/kg for normal subjects and 6.5 N/kg for the "not weak" patients with myositis. Error bars represent + 1 SD from the mean. Abbreviation: F/LBM = force per units of lean body mass.

Patients were then stratified as obviously "weak" or "not weak" according to their mass-normalised force results. For this purpose, "weak" was arbitrarily defined as a mass-normalised MVC lower than 1 SD below the mean of the gender-combined normal group (that is, below 5.1 N/kg), whereas "not weak" was arbitrarily defined as a mass-normalised MVC above 5.1 N/kg (Figure 2).

**Figure 3:**
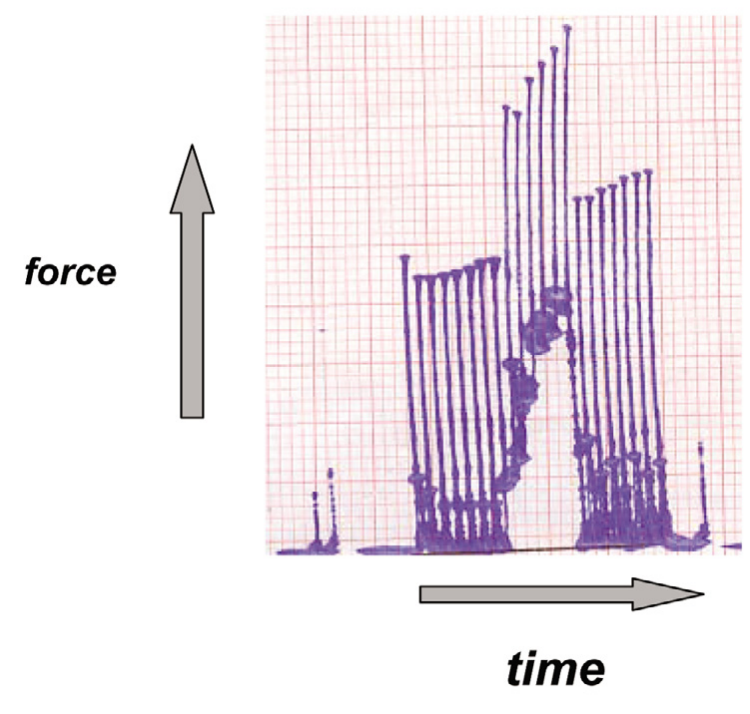
Force tracing of the male patient whose mass-normalised force was the lowest recorded in this study. Large interpolated twitches can be seen on the force trace before, during, and after a supposed maximum voluntary contraction (MVC).

Of the 14 "weak" patients, 12 were female, but the individual with the lowest mass-normalised force was in fact male. When interpolated twitch-status was examined, seven of the 20 patients with myositis demonstrated twitches. Of the six patients stratified as "not weak", one demonstrated twitches, but six of the 14 patients stratified as "weak" demonstrated twitches (Table 3). The largest interpolated twitches were seen in the male individual with the lowest mass-normalised force result (Figure 3).

Of the six "weak" patients displaying interpolated twitches, five had disease designated as active, but the male subject with the largest twitches had disease designated as inactive. The disease duration of those "weak" patients with positive twitches was not significantly different to that of those "weak" patients without twitches, 7.3 (2.7) vs. 5.3 (5.5) years, respectively (*p *> 0.05). In two of the "weak" patients without twitches, QF biopsies had been undertaken during the preceding 6 months to investigate whether active disease was present and had demonstrated end-stage disease only, with marked fibre atrophy and with fatty and fibrosis replacement. Designated disease activity status did not influence normalised strength, with mass-normalised force results of 3.1 (1.8) *vs. *4.3 (3.0) N/kg in active and inactive disease, respectively (*p *> 0.05). Not surprisingly, given that patients' CK results were used in assessing their disease activity status, there was a significant correlation between disease activity status and CK (*r *= 0.704, *p *< 0.01). There was, however, no correlation between CK and CRP (*r *= 0.1) or between disease activity and CRP (*r *= 0.13). In patients, the QF MMT results and those of formal physiological testing by quadriceps chair correlated poorly (for example, patients 6 and 20 both scored 4^+ ^on MMT), but their mass-normalised force results were very different, whereas patients 11, 13, and 16 scored 5 on QF MMT, but all were clearly weak on formal physiological testing.

## Discussion

The detection of interpolated twitches during supposed MVCs in nearly half of the myositis patients designated as "weak" confirms that voluntary activation was incomplete in those subjects. Moreover, given the submaximal stimulation voltages used here, this result may represent an underestimate of the proportion of patients with myositis who do suffer with central activation problems. Given that current QF myalgia excluded patient participation, to minimise the possibility of pain-related inhibition of contraction, these results may suggest that voluntary activation failure had arisen through poor motivation, even though these patients had been pushed as intensely as normal subjects during MVC attempts, with respect to verbal encouragement and visual feedback, in order to maximise activation. Alternatively, twitches would also have been detectable if reflex inhibition of anterior horn cell function had given rise to myogenous weakness.

One might predict that, if T cell infiltration can stimulate afferents to inhibit anterior horn cell function, such inhibition would be most marked where infiltration was greatest. This would fit with the clinical observation that weakness is often worse when infiltration would likely be greatest (that is, before treatment starts in new-onset disease and during disease relapses). It might therefore be speculated that interpolated twitches would more likely be detected in acute or relapsed disease, although this proposition has not been tested. If, however, only a small number of attacking T cells are required to induce reflex inhibition, this could explain the poor correlation observed between a muscle's strength deficit and the degree of its inflammatory cell infiltration at biopsy [6,7]. This is because, in view of their shape, muscle cells have very large surface areas relative to the tiny fraction of which can be sampled at biopsy. Small numbers of T cells could thus be present and functionally relevant (that is, causing inhibition) yet missed at biopsy through simple sampling error. These discussions may, however, represent an oversimplification because, although the largest twitches seen in this study were demonstrated in the weakest patient, this patient's disease was clinically adjudged inactive. Although the twitch interpolation technique seems capable of detecting problems of central activation, it cannot discern between the relative contributions of motivational failure and reflex inhibition (that is, myogenous weakness due to reflex inhibition remains unproven).

If reflex inhibition was confirmed in myositis, what is the explanation for the absence of QF twitches in some "weak" patients with myositis? One possibility is that the inflammatory processes have been fully suppressed by treatment, so there is no inflammatory cell infiltrate to stimulate muscle afferents. Ongoing weakness in this situation would presumably then result from the nonimmune, myositis-induced defects already discussed. Another possibility is that inflammation-induced damage has disrupted the activation processes and/or the contractile apparatus, which thus cannot respond to excitation. That neuropathic EMG features occur in acute myositis [41-43] clearly suggests that coincidental terminal motor efferent, neuromuscular junction and muscle membrane damage can occur in myositis, which would explain excitation failure-induced weakness (that is, twitches would not be inducible if any these defects were present). However, EMGs undertaken in patients with more chronic myositis do not usually show neuropathic features. Where irreversible secondary damage to the contractile apparatus has occurred, including fibre atrophy and loss from fatty and/or fibrous replacement, the resulting contractile failure would also be insurmountable by superimposed twitches. In keeping with these discussions, three of the weak patients without twitches had muscle damage assessed as so severe that independent living was impossible, and two of these had had recent biopsies confirming the presence of end-stage damage, with severe atrophy and fatty and fibrous replacement. The absence of interpolated twitches in weak patients without obvious clinical muscle wasting is clearly in keeping with the growing body of evidence demonstrating that nonimmune mechanisms are involved in weakness induction in myositis [15] and that these mechanisms likely cause weakness by impairing contractile function.

If reflex inhibition does occur in myositis and it is due to afferent stimulation by inflammatory cells, what is the explanation for the detection of interpolated twitches in patients such as the one illustrated in Figure 3 (that is, in patients whose disease is thought inactive)? One possibility is that such disease is not in fact inactive, but the tools used to assess disease activity are simply too crude to detect low level disease. More sensitive tools would clearly be required to test this possibility. The ability to induce interpolated twitches, by surface stimulation over muscle motor points, requires intact function of terminal efferents, neuromuscular junctions, and muscle membranes. Thus, the finding of interpolated twitches in some patients seems to preclude in them the failure of any of these structures as a cause of the voluntary weakness. A potential alternative explanation for the finding of twitches in inactive myositis is that irreversible damage to the afferent apparatus (for example, muscle spindles and intrafusal fibres) occurred before the inflammation resolved. Such "desensitization" might reduce 1a afferent activity, thereby reducing stretch reflex gains and the excitability of the alpha motor neurones and so render descending motor impulses less effective. As long as the terminal efferents, and so on, are intact, muscle motor point stimulation would still induce interpolated twitches. Afferent dysfunction seems an attractive explanation here, if one considers the difficulty experienced by normal but MVC-naïve individuals when trying to produce MVCs without prior familiarisation/feedback. Contractions as vigorous as MVCs are rarely used on a day-to-day basis and so are infrequently perceived. In normal subjects, the MVC familiarisation process allows repeated perception of the sensation and effort of attaining MVCs, which can thereafter be reproduced reliably. In myositis patients with afferent damage, such familiarisation would clearly be more difficult.

The sole aim of the current study was to establish whether central activation failure occurs in patients with myositis. To better understand the detected inability of some patients to produce maximum force with voluntary activation and to better understand the origin(s) of this deficit(s) will require further, detailed neurophysiological investigations using well established techniques. These include the use of H-responses to examine afferent function and transcranial magnetic stimulation to test anterior horn cell excitability. At whatever neurophysiological level such investigations confirm the problem to be, the demonstration that central factors can contribute to weakness induction in myositis is not at odds with the other proposed nonimmune mechanisms already outlined [8-15]. These overall discussions highlight that multifactorial contributions could be made from peripheral and central mechanisms and could have an immune or nonimmune origin. Until more is learnt about the mechanisms of weakness induction in myositis, designing therapies to improve strength and performance will continue to be problematic, a situation compounded by the limitations of the tools currently available for assessing disease activity and damage. The disparity between the results of MMT and formal physiological testing in patients highlights such limitations.

Potential criticisms of this study include the age differences of normal subjects and patients and the relatively small number of patients studied. As pointed out in Materials and methods, the rationale for having a normal group was not to make direct comparisons with patients but instead to generate normative mass-normalised QF MVC results from which patients could arbitrarily be stratified as "weak" or "not weak." The normal subject/patient age difference is also conceded, but it has not impaired the ability to test the study hypothesis. Indeed, if only patients' results had been presented here, proof of hypothesis would still have been provided. The inclusion of normal subjects has, however, improved our ability to discuss the potential cause(s) of the detected central activation problem. With respect to the patient numbers used, the Salford myositis database contains approximately 45 patients with myositis (that is, more than many UK rheumatology units). Even so, some of these patients are old and frail, and some were unable or unwilling to help. Others have coexisting knee joint pathologies precluding their participation, whereas others who agreed to participate were subsequently intolerant of surface stimulation, even at the submaximal voltages used. It is because of the small patient numbers studied that, although central activation failure has been demonstrated, the results presented must be regarded as preliminary and as posing many unanswered neurophysiological questions. Another problem is that no attention has been paid here to the phenomenon of fatigue, which is very common in myositis. All central and peripheral causes of weakness could potentially also cause problems with fatigue, so this is a valid criticism. However, the issue of fatigue was not a study aim, so this issue will have to be addressed in future studies.

## Conclusion

This is the first study to demonstrate that voluntary activation failure does contribute to ongoing muscle weakness in some treated myositis patients. However, myositis-induced weakness appears a multifactorial problem, comprising central activation and peripheral contractile failures and perhaps afferent failure. Large, multicentre studies correlating clinical, neurophysiological, MRI, and histological parameters are now needed to further elucidate the complex issues of weakness induction and fatigue induction in myositis. Such studies will need to include prospective assessments in new-onset patients, if treatment-induced changes in the physiological variables are to be assessed. Improving the treatment of myositis-induced weakness and fatigue will depend on the outcome of such studies.

## Abbreviations

BM = body mass; CK = creatinine kinase; CRP = C-reactive protein; DM = dermatomyositis; EMG = electromyography; LBM = lean body mass; MMT = manual muscle testing; MRI = magnetic resonance imaging; MVC = maximum voluntary contraction; PM = polymyositis; QF = quadriceps femoris

## Competing interests

The authors declare that they have no competing interests.

## Authors' contributions

CBM carried out testing of individuals with myositis and the analysis of results and drafted the manuscript. AOAO carried out testing on the normal controls. KTE coordinated the logistics of the experiments. RGC conceived the study, its design and coordination, carried out the experiments with CBM, and helped draft the manuscript. All authors read and approved the final manuscript.

## Figures and Tables

**Table 1:** Clinical and laboratory features of the 20 patients with idiopathic inflammatory myositis

Patient	Age/gender	IIM Subtype	IIM duration (yr)	Treatment	CK IU/l (24–170)	CRP IU/l (0–10)	MMT QF	EMG	MRI	Disease activity
1	35/M	PM/MCTD	8	P 5, Aza	60	9	5	ND	ND	Inactive
2	34/M	DM	0.5	P 30, SELAM	95	12	5	+	+	Active -
3	46/M	PM/CTD	6	P 20, MMF	109	40	5	ND	ND	Inactive
4	62/F	PM	1	P 7, SELAM	97	14	5	+	ND	Active -
5	59/F	PM	8	-	48	43	5	ND	ND	Inactive
6	62/F	DM	6	-	129	10	4+	ND	ND	Inactive
7	68/F	PM	1	Mtx	653	10	5	+	ND	Active -
8	40/F	DM	6	P 30, CyA, Mtx	486	11	4+	ND	ND	Active -
9	47/M	PM	12	P 10, Mtx	3,500	2	4-	+	+	Active +
10	67/F	PM/MCTD	10	P 10, Mtx	170	2	4+	ND	+	Active
11	58/F	PM/UCTD	1	P 7.5, SELAM	181	2	5	ND	ND	Active
12	50/F	PM	0.5	P 10, CyA, Aza	53	2	4	+	ND	Inactive
13	59/F	DM	7	P 7.5, Aza	144	6	5	ND	ND	Active -
14	66/F	PM	1	P 10, SELAM	159	2	4+	ND	ND	Active
15	43/F	PM/MCTD	3	P 30, CyA, Aza	305	1	4	ND	ND	Active -
16	41/F	PM	8	P 15	282	7	5	ND	+	Active -
17 *	49/F	PM	12	P 7.5	296	22	3-	ND	ND	Active
18	46/F	PM	6	P 30, MMF	3,331	31	3+	ND	ND	Active +
19 *	68/F	PM/MCTD	5	P 7, Mtx	83	8	4	ND	ND	Inactive
20	50/M	DM	10	Mtx	39	2	4+	ND	-	Inactive

CRP and CPK levels were current at the time of recruitment and are those used during disease activity and damage assessments. Extended MMT score is that for dominant QF. If EMG or MRI of QF had been performed within the previous 6 months, - or + indicates the absence or presence of inflammation, respectively. *Patients 17 and 19 had had muscle biopsies within the previous 6 months, and both showed end-stage disease with severe muscle atrophy and fatty and fibrosis replacement. Aza, azathioprine; CK, creatinine kinase; CRP, C-reactive protein; CyA, cyclosporin A; DM, dermatomyositis; EMG, electromyography; IIM, idiopathic inflammatory myositis; IU/l, international units per litre; MCTD, mixed connective tissue disease; MMF, mycophenolate mofetil; MMT, manual muscle testing; MRI, magnetic resonance imaging; Mtx, methotrexate; ND, (test) not done; P, prednisolone (daily dose, mg); PM, polymyositis; QF, quadriceps femoris; SELAM, patient participating in "SEcond Line Agents in Myositis" study, so on prednisolone plus Mtx or placebo and CyA or placebo; UCTD, undifferentiated connective tissue disease.

**Table 2:** Myositis disease activity and damage scores

Score	Disease activity status (weakness attributable to activity)	Score	Disease damage status (weakness attributable to damage)
Inactive	No disease activity. Dose decrements contemplated or actioned	0	No weakness.
Active -	Mild activity, but stable. No dose increments contemplated, decrements contemplated or actioned.	1	Mild persisting weakness, but normal ADL and/or occupation.
Active	Moderate activity. Dose increments contemplated or actioned.	2	Obvious persisting weakness. Limited ADL and/or occupation.
Active +	Severe activity. Additional agents contemplated or actioned.	3	Severe persisting weakness. Unable to function independently.

Patients' disease activity levels were scored on "intention-to-treat" principles. ADL, activities of daily living.

**Table 3:** The muscle strength, disease activity and damage scores, and twitch interpolation results of patients with myositis

QF (MVC) Strength category	Patient number	QF mass-normalised force F/LBM (N/kg)	Activity status	Damage score (0–3)	Twitch status (-/+)
"Not weak"	1	8.83	Inactive	0	-
	2	7.17	Active -	1	-
	4	5.64	Active -	1	-
	5	5.63	Inactive	1	-
	6	5.40	Inactive	1	-
	3	6.40	Inactive	2	+
"Weak"	7	4.14	Active -	1	-
	8	3.97	Active -	1	-
	9	3.69	Active	1	-
	11	2.82	Active	1	-
	12	2.28	Inactive	3	-
	14	2.10	Active	1	-
	*17	1.30	Active	3	-
	*19	0.99	Inactive	3	-
	10	3.32	Active	1	+
	13	2.11	Active -	2	+
	15	1.84	Active -	3	+
	16	1.76	Active -	1	+
	18	1.00	Active +	2	+
	20	0.80	Inactive	3	+

Twenty patients with myositis listed according to their mass-normalised strength and twitch interpolation results, and stratified as "not weak" and "weak" according to their F/LBM results (see text). In general terms, as QF strength decreased, the incidence of positive twitches increased. Cases 17 and 19 had had muscle biopsies within the previous 6 months, and the results showed end-stage disease with severe muscle atrophy and fibrosis. F/LBM, force per units of lean body mass; MVC, maximum voluntary contraction; QF, quadriceps femoris.
